# Lipoxin Receptors

**DOI:** 10.1100/tsw.2007.186

**Published:** 2007-09-01

**Authors:** Mario Romano, Irene Recchia, Antonio Recchiuti

**Affiliations:** Department of Biomedical Sciences, Aging Research Center, Ce.S.I., “Gabriele D'Annunzio” University Foundation, Via dei Vestini, 31 66013, Chieti, Italy

**Keywords:** arachidonic acid, lipoxin, leukotriene, inflammation, anti-inflammatory eicosanoids, receptor, signaling

## Abstract

Lipoxins (LXs) represent a class of arachidonic acid (AA) metabolites that carry potent immunoregulatory and anti-inflammatory properties, LXA_4_ and LXB_4_ being the main components of this series. LXs are generated by cooperation between 5-lipoxygenase (LO) and 12- or 15-LO during cell-cell interactions or by single cell types. LX epimers at carbon 15, the 15-epi-LXs, are formed by aspirin-acetylated cyclooxygenase-2 (COX-2) in cooperation with 5-LO. 15-epi-LXA_4_ is also termed aspirin-triggered LX (ATL). *In vivo* studies with stable LX and ATL analogs have established that these eicosanoids possess potent anti-inflammatory activities. A LXA_4_ receptor has been cloned. It belongs to the family of chemotactic receptors and clusters with formyl peptide receptors on chromosome 19. Therefore, it was initially denominated formyl peptide receptor like 1 (FPRL1). This receptor binds with high affinity and stereoselectivity LXA_4_ and ATL. It also recognizes a variety of peptides, synthetic, endogenously generated, or disease associated, but with lower affinity compared to LXA_4_. For this reason, this receptor has been renamed ALX. This review summarizes the current knowledge on ALX expression, signaling, and potential pathophysiological role. The involvement of additional recognition sites in LX bioactions is also discussed.

## LIPOXINS

### Biosynthesis

Lipoxins (LXs) represent a class of arachidonic acid (AA) metabolites with unique anti-inflammatory and immunoregulatory properties. The main components of this series are $LXA_4$ (5*S*,6*R*,15*S*-trihydroxy-7,9,13-*trans*-11-*cis*-eicosatetraenoic acid) and its positional isomer $LXB_4$ (5*S*,14*R*,15*S*-trihydroxy-6,10,12-*trans*-8-*cis*- eicosatetraenoic acid). In human cells, LX formation occurs mainly via transcellular metabolism, although biosynthesis in single cell types has been reported. Two main routes of LX formation have been described. One involves transcellular exchanges between polymorphonuclear leukocytes (PMNs) and platelets. Receptor-mediated activation of these cell types yields significant accumulation of $LXA_4$ and $LXB_4$[1]. This route proceeds through conversion of the 5-LO product leukotriene (LT) $A_4$, derived from activated PMNs, into LXs by the platelet 12-LO[2]. This enzyme abstracts hydrogen from carbon-13 of $LTA_4$ and inserts molecular oxygen at carbon-15 to yield a delocalized cation. The cation is attacked by water either at carbon-6 to give $LXA_4$ or at carbon-14 to give $LXB_4$[3] (Fig. 1). Thus, platelet 12-LO has LX synthase activity[4]. Indeed, studies with recombinant platelet 12-LO showed that this enzyme has a high affinity for $LTA_4$, comparable to that calculated for AA (apparent $K_m = 7.9 \pm 0.8$ and $6.2 \pm 1.8$ μ*M*, respectively), indicating that LX synthase may represent a major activity of platelet 12-LO[4]. *In vivo*, this pathway is likely to occur during coronary angioplasty[5] and following strenuous exercise[6], when interactions between PMNs and platelets have been documented.

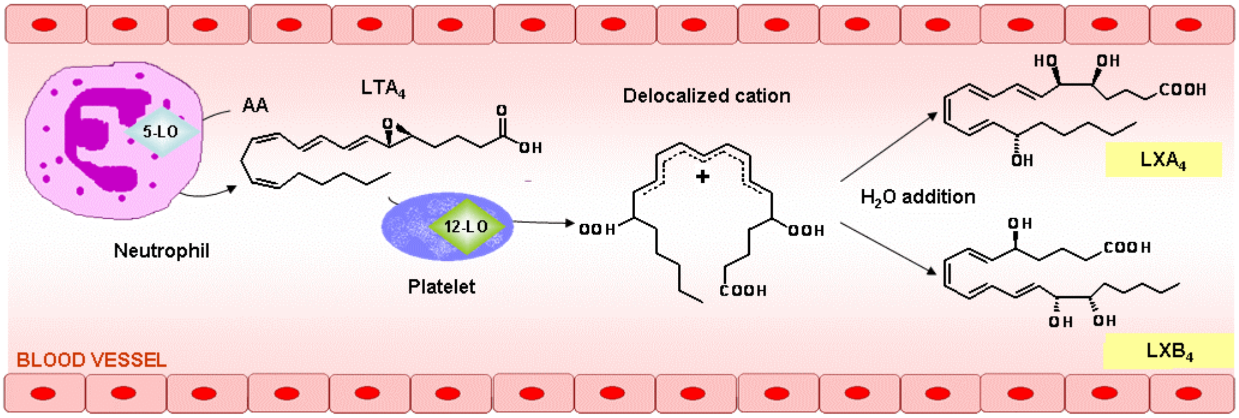

**FIGURE 1.** The 5-/12-LO biosynthetic pathway of LXs (see text for details).

A second route of LX biosynthesis involves the cooperation between epithelial cells and PMNs[7]. The enzymes involved in this pathway are 15- and 5-LO. AA is initially oxygenated by 15-LO to yield 15*S*-hydroxytetraenoic acid (15*S*-HETE). This is converted by 5-LO to a 5*S*,6*S*,15*S*-epoxytetraene intermediate, which is enzymatically transformed into $LXA_4$ and $LXB_4$ (Fig. 2). Alternatively, $LTA_4$ can be converted by 15-LO to the 5*S*,6*S*,15*S*-epoxytetraene and then to LXs. LX generation by this route also occurs in isolated human PMNs[8], eosinophils[9], alveolar macrophages[10], as well as during coincubations of PMNs and eosinophils[9]. LX generation in the respiratory tract may be mainly generated by this pathway[11].

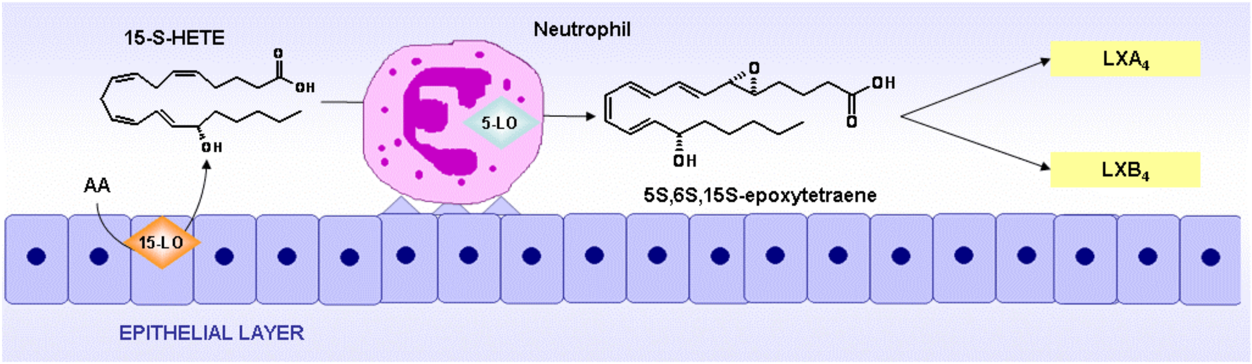

**FIGURE 2**. The 5-/15-LO pathway of LX biosynthesis (see text for details).

A distinct biosynthetic route yields an additional class of LXs, the 15*R* epimers, also termed 15-epi-LXs. They are generated by aspirin-acetylated cyclooxygenase-2 (COX-2) in cooperation with 5-LO.

COX-2, acetylated by aspirin, metabolizes AA to 15*R*-HETE, instead of prostanoids. 15*R*-HETE is then taken up by PMNs and converted to 15*R*-$LXA_4$, also termed aspirin-triggered lipoxin (ATL), and 15*R*-$LXB_4$ by a 5-LO-catalyzed reaction[12]. Biosynthesis of 15-epi-LXs occurs during transcellular exchanges between aspirin-treated endothelial cells and PMNs[12] (Fig. 3), coincubations of rat hepatocytes with nonparenchymal liver cells[13], or of A549 cells and PMNs[14]. Evidence of 15-epi-LX formation *in vivo* has been obtained by measuring urinary levels of ATL in healthy volunteers subjected to aspirin (100 mg/die) treatment for at least 8 days[15]. Also, ATL plasma levels were measured in healthy subjects taking low-dose aspirin daily for 8 weeks[16].

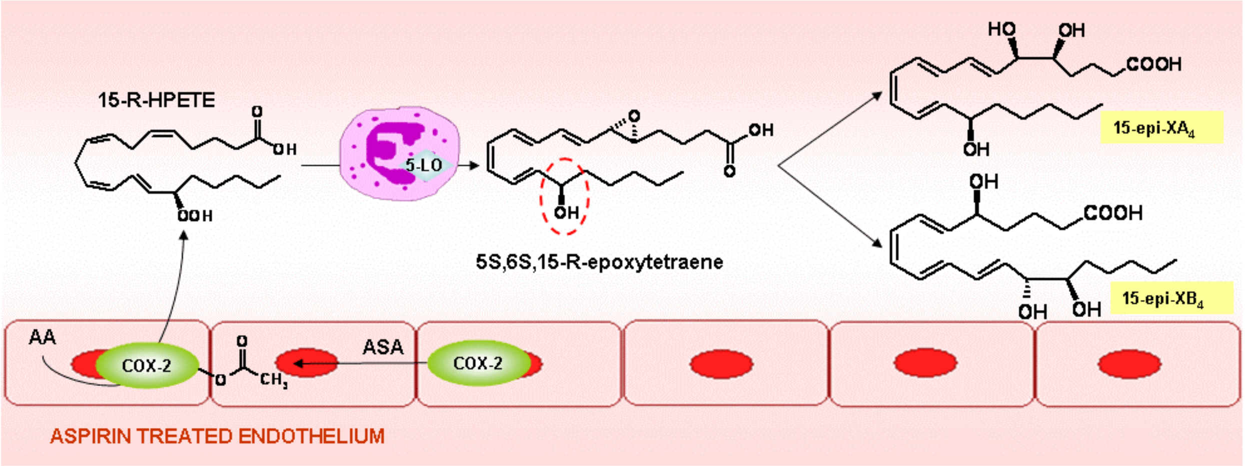

**FIGURE 3**. Scheme of 15-epi-LXs biosynthesis (see text for details).

## Bioactions

LXs and epi-LXs are potent regulators of the immune-inflammatory response. A significant contribution for the understanding of their bioactions derives from the synthesis of stable analogs. In fact, native compounds are rapidly metabolized and inactivated, mainly by monocyte/macrophage. $LXA_4$ is dehydrogenated by 15-hydroxyprostaglandin dehydrogenase (15-PGDH) to yield 15-oxo-$LXA_4$. This is reduced by 15-oxoprostaglandin 13-reductase, also known as $LTB_4$ 12-hydroxydehydrogenase (PGR/$LTB_4$DH), to form 13,14-dihydro-15-oxo-$LXA_4$, which is converted by 15-PGDH into 13-14-dihydro-$LXA_4$. $LXB_4$ follows a similar metabolic pattern, being converted to 5-oxo-$LXB_4$, 5-oxo-6,7-dihydro-$LXB_4$, and to 6,7-dihydro-$LXB_4$. $\omega$-Oxidation of LXs has been also reported[17].

A number of LX and epi-LX stable analogs have been synthesized[17]. Among these, 15-epi-16-(para-fluoro)-phenoxy-$LXA_4$ (ATLa) has been widely used *in vivo* and *in vitro*. Notably, the 3-oxa derivatives of ATLa display enhanced chemical stability due to a marked resistance to beta oxidation[18]. These analogs, as well as ATLa, displayed a potent anti-inflammatory activity *in vivo*, even after oral administration, indicating that they may be of therapeutic utility in inflammatory diseases[19].

## Cellular Targets

LX and ATL, at nanomolar concentrations, exert potent counter-regulatory actions on cells involved in the immune-inflammatory response. In particular, LX and ATL ***inhibit*** PMN functions, such as chemotaxis[20], tumor necrosis factor (TNF)-α–induced superoxide anion generation, interleukin (IL)-1β release and expression[21], lipopolysaccharide (LPS)-induced IL-8 expression and release[22], homotypic aggregation[23], azurophilic degranulation[24], CD11/CD18 expression[23], epithelial and endothelial transmigration[25,26], and nuclear-factor kappa beta (NF-κB) activation[22]. On the other hand, LX and

ATL ***stimulate*** a number of monocyte/macrophage responses, i.e., chemotaxis[27], calcium mobilization[28], and nonphlogistic phagocytosis of apoptotic PMNs[29]. In monocytes, $LXA_4$ and ATL also reduce LPS-stimulated peroxynitrite formation, IL-8 expression and release, and NF-κB activation[22]. Thus, $LXA_4$ and ATL appear to down-regulate the mounting of inflammatory responses and to up-regulate mechanisms related to the resolution of inflammation. However, these compounds may play a more complex role in the modulation of the immune response. Early data documented the inhibition of NK-dependent cytotoxicity by $LXA_4$[30]. More recent results show that ATLa can block TNF-α secretion from anti-CD3–stimulated T lymphocytes[31]. Furthermore, $LXA_4$ regulates dendritic cell migration, IL-12 production[32], and expression of suppressor of cytokine signaling (SOCS)-2[33], thus contributing to the antimicrobial response. Along these lines, $LXA_4$ inhibits migration and degranulation of eosinophils *in vitro*[34] as well as allergen-induced eosinophil trafficking *in vivo*[35]. Notably, LXs reduce P-selectin expression in endothelial cell, thus controlling the initial phase of leukocyte recruitment to inflammatory sites[36]. In endothelial cells, nanomolar concentrations of ATLa up-regulated the expression of heme oxygenase-1, which is recognized as a main anti-inflammatory pathway[37]. Likewise, $LXA_4$ amplified heme oxygenase-1 expression in corneal epithelial cells[38]. $LXA_4$ also regulates fibroblast functions relevant for tissue remodeling in inflammation. In particular, it inhibits IL-1β–induced production of matrix metalloproteinase (MMP)-3 at nanomolar concentrations, while it increases expression of tissue inhibitor of metalloproteinase (TIMP)-1 and 2[39]. Likewise, $LXA_4$ exerts counteregulatory actions on key inflammatory circuits, namely, peroxisome proliferator-activated receptor-α and CINC-1 in hepatocytes[40]. Moreover, it antagonizes *Pseudomonas aeruginosa*– or TNF-α–induced IL-8 secretion in human epithelial respiratory cells[41,42], highlighting the potential beneficial activity of $LXA_4$ in cystic fibrosis and acid lung injury (LX and ATL cellular actions are summarized in Table 1).

## LX in Disease

Results with a variety of animal models of disease have documented potent anti-inflammatory, proresolution activities of LX and ATL *in vivo*. In the respiratory tract, ATLa reduced methacolin-stimulated broncoconstriction of mice sensitized with ovoalbumin[43]. In this model, it also inhibited leukocyte infiltration, as well as release of $T_H2$ cytokines. ATLa at subnanomolar/nanomolar concentrations reduced the airway inflammatory response evoked by *P. aeruginosa* in mice[41]. This may be relevant in cystic fibrosis, where *P. aeruginosa* colonization is frequent. Indeed, reduced $LXA_4$ levels were found in broncoalveolar lavage (BAL) fluids from patients with cystic fibrosis[41]. The gastrointestinal district represents another preferential target of LX and ATL *in vivo*. $LXA_4$ protects from aspirin-induced gastric damage in rat[44]. ATLa reduced inflammatory infiltration and protein extravasation in rat peritonitis[45]. In addition, the 3-oxa-ATL analog attenuated the inflammatory reaction in hapten-induced colitis[46], and oral administration to mouse of ATLa at 10 μg/day limited weight loss, hematochezia, and mortality in dextran sodium sulfate–induced colitis[47]. LX and ATL may be beneficial in periodontal disease, as topical application of ATLa dramatically reduced bone destruction and local inflammation in a rabbit model of periodontitis[48]. Topical ATLa also inhibited cutaneous inflammation induced by a variety of inflammatory agents in mouse and guinea pig, although it was less potent than methylprednisolone aceponate[49]. In the mouse eye, topical application of 1 μg $LXA_4$ or ATLa promoted corneal re-epithelialization after thermal injury or de-epithelialization induced using a corneal rust ring remover, and modulated host-defense mechanisms[50]. Also, treatment of donor bone marrow cells with 100 ng/ml ATLa and i.v. administration of 50 μg/kg ATLa to recipient mouse delayed death by graft-vs.-host disease (GvHD)[51]. This treatment attenuated GvHD-related symptoms, namely, diarrhea, skin lesions, weight loss, and eye inflammation[51]. Thus, results with animal models of disease are encouraging and support the hypothesis that stop signals of inflammation, as LXs and ATL, may be of therapeutic use in human disease (Fig. 4).

**TABLE 1**
**$LXA_4$ and ATL Most Relevant Bioactions in ALX-Expressing Cells**

| Cell Type | | Bioactions |
|---|---|---|
|  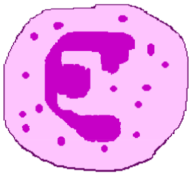 | Neutrophils | ↓ Chemotaxis, adhesion, transmigration<br>↓ Degranulation, IL-1β, IL-8, and $O_2^-$ production<br>↓ CD11b/CD18 expression<br>↓ Adhesion to endothelium<br>↓ Homotypic aggregation |
|  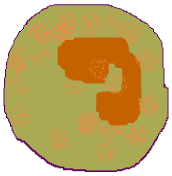 | Eosinophils | ↓ Migration and degranulation<br>↓ Eotaxin and IL-15 production |
|  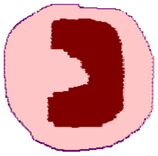 | Monocytes/macrophages | ↑ Chemotaxis and adhesion to laminin<br>↑ Nonpholgistic phagocytosis of apoptotic PMNs<br>↓ Peroxynitrite formation<br>↓ IL-8 production<br>↓ NF-κB activation |
|  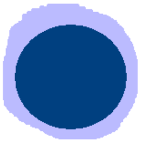 | T lymphocytes | ↓ TNF-α secretion |
|  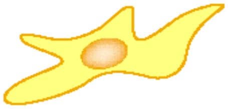 | Dendritic cells | ↓ IL-12 production<br>↑ SOCS-2 expression |
|  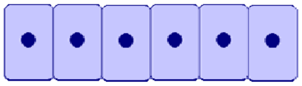 | Epithelial cells | ↓ IL-8 release |
|  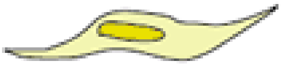 | Fibroblasts | ↓ CTGF-induced proliferation<br>↓ IL-6, IL-8, and MMP-3 production<br>↑ TIMP-1 |
|  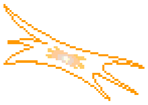 | Renal mesangial cells | ↓ PDGF- and $LTD_4$-induced proliferation |

**TABLE 2 (continued)**

| | | |
|---|---|---|
|  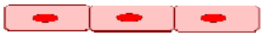 | Endothelial cells | ↑ TF expression<br>↑ $PGI_2$ formation<br>↓ VEGF-induced proliferation, adhesion, and migration<br>↓ P-selectin expression<br>↑ Heme oxygenase-1 expression |
|  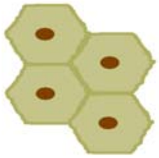 | Hepatocytes | ↓ PPARα and CINC-1 expression |

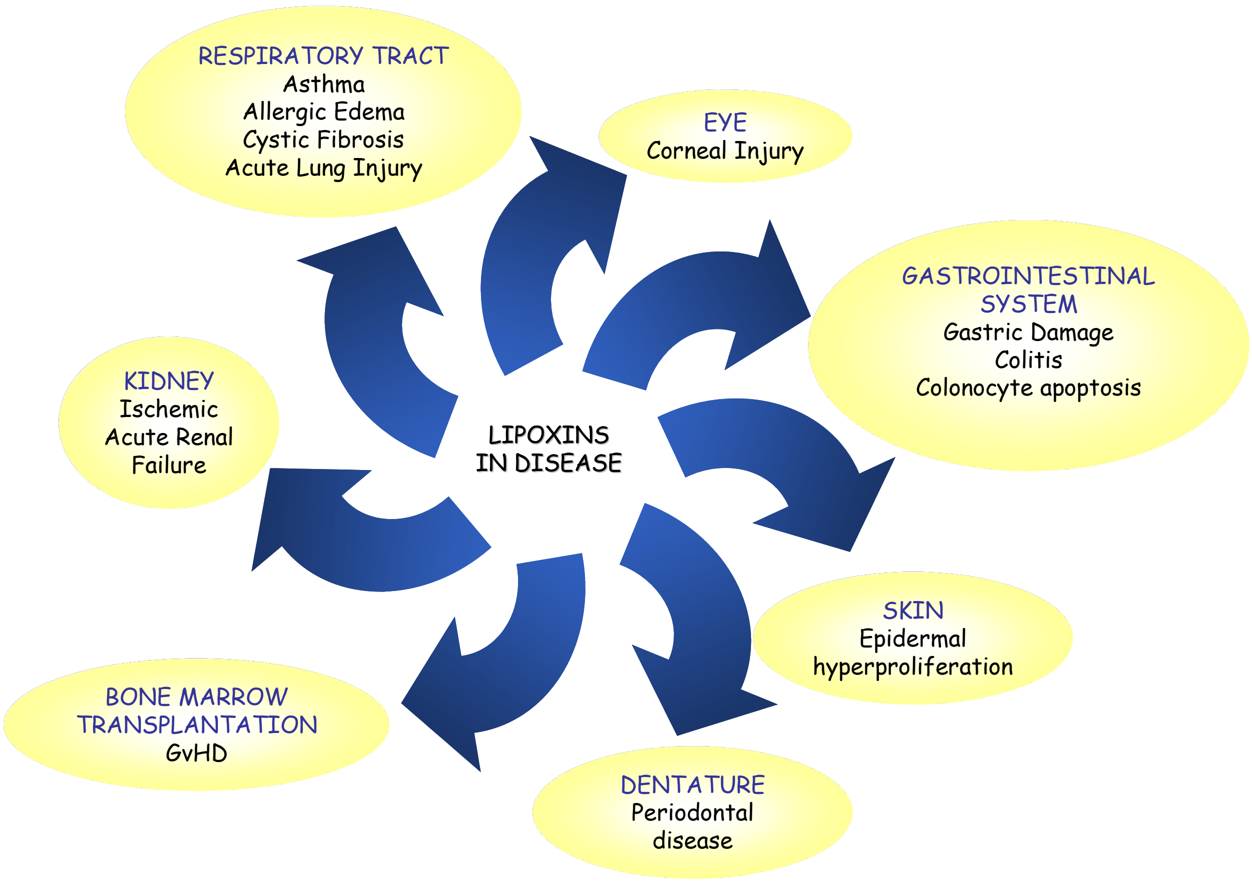

**FIGURE 4**. LX in human disease (see text for details).

## ALX, THE $LXA_4$ RECEPTOR

### Identification

To elucidate LX interaction with target cells and intracellular signaling, binding studies with radiolabeled [11,12-$^3$H-$LXA_4$] were carried out. Specific binding sites were initially recognized in PMNs ($K_d$ = ~0.5 n*M*; ~1,800 sites/cell)[52]; in HL-60 cells differentiated with phorbol 12-myristate 13-acetate (PMA), retinoic acid, or dimethyl sulfoxide (DMSO) ($K_d$ = ~0.6 n*M*); and in endothelial cells ($K_d$ = ~11 n*M*)[53]. Interestingly, [11,12-$^3$H-$LXA_4$] binding to endothelial cells was displaced by excess $LTD_4$ or by the $LTD_4$ receptor antagonist SKF 104353, indicating that $LXA_4$ may interact with a $LTD_4$ receptor in endothelial cells[53]. This observation was later confirmed by Gronert et al.[54], who showed that [$^3$H]-ATLa binds to recombinant cysteinyl leukotriene receptor ($CysLT_1$) with equal affinity of $LTD_4$, but it antagonizes $LTD_4$-induced proinflammatory signaling.

The nature of the $LXA_4$ specific binding site in PMNs remained elusive, however, although the observation that some intracellular signaling events triggered by $LXA_4$ were pertussis toxin (PTX) sensitive and that the binding of radiolabeled $LXA_4$ with PMN granule membrane fractions was regulated by GTPγS[52] suggested that a G-protein coupled recognition site was involved in $LXA_4$ signaling. A major breakthrough in the identification of the $LXA_4$ receptor came from the screening for [11,12-$^3$H-$LXA_4$] binding to orphan G-protein coupled receptors (GPCRs). One plasmid DNA, initially denominated pINF114, conferred to transfected CHO cells specific $^3$H-$LXA_4$ binding, with an apparent $K_d$ of ~1.7 n*M*[53]. $LXA_4$ induced GTP hydrolysis and AA release in transfected cells, and these responses were PTX sensitive[55]. pINF114 was therefore *deorphanized* and recognized as a $LXA_4$ receptor. A number of independent studies reported the cloning of this receptor, attributing to it a variety of different names. Its sequence is high homologous (69%) with that of the formyl peptide receptor (FPR) and, therefore, it was termed formyl peptide receptor like 1 (FPRL1) by Murphy et al.[56]. It was also named FPRH1[57], FPR2[58], RFP[59], and HM63[60]. The more recent nomenclature of the International Union of Pharmacology denominated this receptor ALX, in view of the evidence that $LXA_4$ is the most potent endogenous agonist for this receptor[61]. Notably, $LXA_4$ recognition of ALX is highly stereoselective. For binding and activity, a 15-hydroxyl group, the tetraene structure, the 11,12-*cis*, and the 13,14-double bond within the tetraene are required. The lines of evidence that justify the attribution of the name ALX to this receptor are reviewed in Chiang et al.[62].

### The FPRL1/ALX Gene in Human and Mouse

The human FPRL1/ALX gene maps on chromosome 19 at 19q13.3-q13.4[57]. It encompasses 9.60 kb on the direct strand. The gene contains two exons and two gt-ag introns. Alternative splicing produces two different transcripts, 2181 and 2636 bp long, respectively, encoding one protein product. In the mRNA, the 5'UTR encompasses ~773 bp, whereas the 3'UTR contains ~803 bp followed by the polyA. A variant polyadenylation signal ATTAAA is located ~21 bp before the polyA. In one population study with blood donors in North America, no polymorphic variants within the coding reading frame of FPRL1/ALX were detected[63]. The human FPRL1/ALX protein contains 351 residues for a calculated MW of 38.9 kDa, arranged in seven putative transmembrane domains (TMs) with the N-terminus placed on the external side and the C-terminus on the intracellular side. Thus, ALX belongs to the GPCR family.

Human FPRL1/ALX is a member of the FPR gene cluster (FPR, FPRL1, FPRL2) that spans ~80 kb on chromosome 19. FPRL1/ALX displays 69% amino acid sequence homology with FPR and 83% with FPRL2. The mouse ALX receptor was cloned by Takano et al. utilizing a spleen cDNA library[64]. This receptor, termed *Lxa4r/Fprl1*, bound radiolabeled $LXA_4$ with high affinity ($K_d$ ~1.5 n*M*) in CHO-transfected cells and transduced anti-inflammatory responses. It is highly expressed in PMNs and its amino acid sequence is 73% identical with the human ALX. Utilizing probes for the human genes, Gao et al. cloned six mouse genes, which form a cluster on mouse chromosome 17 and were denominated *Fpr1*

and *Fpr-rs1-5*[65]. Of these genes, *Fpr-rs1* and *Fpr-rs2* displayed the highest homology with FPRL1/ALX. In a separate study, Vaughn et al. identified a clone denominated 8C10, which is highly homologous to *Lxa4r/Fprl1* (89% identity) and identical to *Fpr-rs2*[66]. COS-1 cells cotransfected with 8C10 and Gα16 cDNA displayed inositol-1,4,5-triphosphate ($IP_3$) accumulation when exposed to nanomolar concentrations of $LXA_4$.

A rat $LXA_4$ receptor was recently cloned[45]. It is expressed in leukocytes, lung, and kidney, and its amino acid sequence is 74% homologous with that of human ALX and 84% homologous with that of the mouse $LXA_4$ receptor. The rat $LXA_4$ receptor showed specific binding with $[^3H]LXA_4$ ($K_d$ ~5 n*M*) and inhibited TNF-α–induced NF-κB activity[45].

Thus, a $LXA_4$ receptor, bearing regulatory functions of the immune-inflammatory response, is maintained across species. The preparation of a transgenic mouse model, engineered for targeted overexpression of human (h)ALX in myeloid cells, greatly contributed to the understanding of the functional role of ALX *in vivo*[67]. The hALX mice displayed a ~80% reduction in PMN infiltration in ear skin after challenging with $LTB_4$ plus prostaglandin $(PG)E_2$ and a marked reduction in peritoneal PMN infiltrates in response to zymosan. Remarkably, these responses were observed in the absence of administration of ALX agonists[67]. This observation supports the concept of a predominant anti-inflammatory function of ALX *in vivo*.

## Functional Domains

Site-directed mutagenesis of putative phosphorylation residues Ser-236, Ser-237, and Tyr-302, determined sustained phospholipase $A_2$ and D activation by $LXA_4$ in CHO-transfected cells, as opposed to the transient activation observed with cells transfected with wild-type ALX[68]. Additional information on $LXA_4$ recognition domains derives from chimeric constructs. In particular, studies with chimeras ALX/$LTB_4$ receptor 1 ($BLT_1$) showed that the seventh TM domain and adjacent regions are relevant for $LXA_4$ binding and activity[69]. Domains involved in peptide signaling (see section on peptidic ligands) have been also identified. Studies with ALX/FPR chimeras localized a number of such domains within the sixth TM domain and the third extracellular loop[70]. Moreover, interaction sites with a 42 amino acid amyloid β peptide ($Aβ_{42}$) were localized at the N-terminus and within a stretch between the fourth TM domain and the third intracellular loop[70]. On the other hand, binding of peptides MMK-1 and MHC required extracellular loops[69]. Thus, distinct domains appear to be involved in the recognition of $LXA_4$ vs. other peptide ligands. This observation may have relevant implications, as ALX activation by peptides can switch responses from anti- to proinflammatory.

## Cellular and Tissue Expression

Initially localized in myeloid cells, ALX expression has been observed in a variety of cell types. In particular, PMNs[52], monocytes[71], basophils[72], dendritic cells[73], and T lymphocytes[31] all express ALX, underscoring the pre-eminent role of ALX in the immune-inflammatory response. On the other hand, human intestinal epithelial cells express the full-length sequence of neutrophil ALX[74]. In these cells, ALX mRNA expression was up-regulated by a number of cytokines, IL-13 (10 ng/ml) and interferon (IFN)-γ (1,000 U/ml) being the most potent. Likewise, IL-1β stimulated accumulation of ALX mRNA in human synovial fibroblasts[39]. In addition, $PGE_2$, generated by acid injury, up-regulated ALX expression in human respiratory epithelial cells[42]. These observations suggest that ALX expression could be transcriptionally regulated, although more direct evidence of ALX transcriptional events is awaited. Interestingly, ALX expression in astrocytoma[75], neuroblastoma[76], and colon cancer cells[74] has been reported. Accordingly, ALX expression in breast, prostate, and ovarian cancer cell lines has been observed (Recchia et al., unpublished observation). These findings suggest that ALX could play a role in cancer proliferation and/or invasion. Finally, ALX appears to be expressed in endothelial cells[77] and in vascular smooth muscle cells (Recchiuti et al., unpublished observation). Thus, ALX involvement in vascular biology needs to be further elucidated.

In relation to organ distribution of ALX, spleen and lung show high abundance of ALX transcript. Lower levels were observed in heart, liver, and placenta[55]. Cellular expression of ALX is summarized in Table 1.

## Peptidic Ligands

Since its identification as the $LXA_4$ receptor, FPRL1/ALX has been proposed as the receptor for a number of peptides, either of viral/bacterial origin or endogenously generated. Peptide and $LXA_4$ signaling may be different, accounting for different bioaction profiles. It has been proposed that this feature, i.e., one receptor structure for multiple ligands and functions, could represent a sort of "genomic economy" within the immune system[62]. How this "*promiscuous selectivity*" is achieved remains a crucial issue. Relevant information regarding this point can be found in an elegant study by Chiang et al.[69]. These investigators generated chimeric receptors with sequences from ALX and from $BLT_1$. These constructs revealed that N-glycosylation is essential for ALX recognition of peptide ligands, but not of $LXA_4$. Moreover, the third extracellular loop, the seventh TM domain, and COOH terminus of ALX are required for $LXA_4$ binding, whereas high-affinity binding of peptide ligands needs additional regions[69]**.**

It is noteworthy that the majority of the ALX peptide agonists appear to trigger proinflammatory signaling (see section on signaling), although their biological role is not completely clear.

***Annexin 1 (ANXA1)*** represents an exception to this pattern. A 37-kDa protein, originally described as lipocortin, ANXA1 is a potent phospholipase $(PL)A_2$ inhibitor. It has been recently reported that ANXA1 and its related peptide Ac2-26 bind to ALX to limit PMN infiltration *in vivo*[78]. A synergistic effect with ATLa was observed, underscoring the control of more than one anti-inflammatory/proresolution pathway by ALX. Moreover, it has been recently shown that annexin-1 and peptide derivatives are released by apoptotic cells (human PMNs, Jurkatt T lymphocytes, human mesangial cells) to promote phagocytosis of apoptotic PMNs by macrophages, likely via ALX[79]

The coagulation/fibrinolytic system is a relevant component of the immune-inflammatory response. ***Urokinase plasminogen activator (uPA)*** is a serine protease that activates plasminogen to plasmin and binds with high affinity to a membrane receptor, uPAR (CD87)[80]. Recent studies indicate that uPAR not only functions as a proteinase receptor that facilitates activation of uPA and focuses proteolysis to the cell surface, but also affects migration, adhesion, differentiation, and growth through intracellular signaling[81]. uPAR is formed by three extracellular domains (D1, D2, and D3) and is anchored to the plasma membrane via glycosylphosphatidylinositol. Because uPAR lacks an intracellular domain, the existence of a transmembrane transducer was hypothesized. This transducer was identified as ALX. In fact, a soluble form of uPAR (termed $D2D3_{88\text{-}274}$) bound to ALX and was displaced by a stable $LXA_4$ analog[82]. $D2D3_{88\text{-}274}$ stimulated cellular chemotaxis *via* ALX. Recently, Mazzieri et al. showed that uPAR requires both integrins and ALX to induce chemotaxis[83]. These findings suggest that ALX may stand at the intersection of different regulatory circuits of the immune-inflammatory response.

The discovery that polypeptides associated with neurodegenerative disorders recognize ALX with affinity ranging from nanomolar to micromolar concentrations has fostered interest in this receptor by investigators from this field.

***Serum amyloid A (SAA)*** is an acute-phase protein whose levels are elevated under chronic or recurrent diseases. On enzymatic cleavage by monocytes and macrophages, SAA can generate amyloidogenic fragments and then amorphous fibrillary deposits, leading to amyloidosis[84]. SAA binds to ALX ($K_d$ = 45 n*M*), stimulates chemotaxis of mononucleated phagocytes and PMNs[85], IL-8 secretion *via* NF-$\kappa$B, extracellular signaling regulated kinase (ERK) 1/2, and p38 MAPK activation[86].

The $\boldsymbol{A\beta_{42}}$ protein, the fragment of the amyloid precursor protein that represents the major component of senile plaques in Alzheimer's disease (AD) patients, also binds and activates ALX and its mouse counterpart, FPR2, inducing chemotaxis and production of oxygen reactive species in mononuclear phagocytes and microglial cells[87]. Notably, ALX-A$\beta_{42}$ complexes are internalized in macrophages leading to formation of fibrils[88].

The short, 21 amino acid, fragment ***PrP$_{106-126}$*** of human prion protein also forms fibrils *in vitro* and stimulates release of proinflammatory cytokines in monocytes and microglia[89]. Le et al. reported that PrP$_{106-126}$ stimulates release of proinflammatory cytokines from monocytes through activation of ALX[90]. On the other hand, ALX has been identified as the receptor for the neuroprotective peptide, ***humanin (HN)***, a 24 amino acid polypeptide, encoded by a gene cloned from an apparent normal region of the AD brain[91]. ALX activation by HN results in suppression of A$\beta_{42}$-induced cytopathic effects in neuroblast cells[76]. Collectively, these data suggest that ALX may have a complex role in amyloidosis and that selective stimulation of the anti-inflammatory signaling of ALX may be beneficial in this setting.

Additional endogenously generated peptides bind and activate ALX; in particular, the ***MHC binding peptide***, which derives from mitochondrial NADH dehydrogenase subunit 1 and binds ALX with high affinity ($EC_{50}$ < 1 n*M*). This peptide is a potent necrosis inducer, and stimulates neutrophil chemotaxis[69] and macrophage-mediated phagocytosis of PMNs[92]. Antimicrobial peptides represent an important component of the innate immune system, being involved in the regulation of the inflammatory response. ***LL37*** is a cleavage fragment of neutrophil cathelidicidin, which is released into injured tissues. It stimulates angiogenesis, promoting wound neovascularization through activation of ALX on endothelial cells[77]. Notably, ATLa inhibited vascular endothelial growth factor (VEGF)-induced neovascularization in a granuloma *in vivo* model[93]. Thus, multiple signals of the immune-inflammatory-related neoangiogenic response appear to converge on ALX.

A novel N-terminally truncated form of β-chemokine, ***CK β 8-1***, was also found to bind ALX, and induced $[Ca^{2+}]_i$ and chemotaxis in PMNs[94]. Interestingly, ***temporin A,*** a natural antimicrobial peptide isolated from the skin secretions of *Rana temporaria*, recognizes ALX, inducing recruitment of PMNs and monocyte/macrophages in mice *in vivo*[95]. This observation lends further support to the concept that ALX is a key regulator of the trafficking of immune-inflammatory cells during antimicrobial host-response.

***Pathogens-derived peptides*** appear to interact with ALX in the micromolar range of $EC_{50}$; among these, some HIV-1 envelope proteins (e.g., T20, T21, N36, V3, and F peptide), bacterial proteins (Hp2-20 from *Helicobacter pylori*), and N-formyl peptides (fMLF)[96,97]. The biological significance of such interactions remains to be fully understood.

The early assumption that formyl group at N-terminus of peptidic sequence was essential for optimal receptor recognition has been challenged by the finding that a large number of ***synthetic peptides***, screened from a random library, are able to activate ALX (and, in same cases, FPR). For instance, WKYMVm and MMK-1 are very potent activators of ALX ($EC_{50}$ in the nanomolar range). On the other side, peptides that antagonize ALX signaling have been identified; in particular, WRW blocked at micromolar concentrations, $[Ca^{2+}]_i$ mobilization induced by WKYMVm, MMK-1, A$\beta_{42}$, as well as superoxide generation and chemotactic migration in PMNs exposed to A$\beta_{42}$[98]. This peptide also inhibited A$\beta_{42}$ internalization in human macrophages. Characteristics of ALX ligands are summarized in Table 2

## Other Nonpeptidic ALX Agonists

Using a cell-based assay in a high-throughput screening, a pyrazolone derivative with potent ALX agonist activity was identified[99]. This compound showed anti-inflammatory activity in a mouse model either by topical application or oral administration, although it was less potent than 16-phenoxy-$LXA_4$ or dexamethasone. This finding opens a new avenue of pharmacological investigation for the synthesis of more potent ALX agonists with anti-inflammatory activity.

## Signaling

Accumulating evidence indicates that ALX signaling is cell and agonist specific. This is not surprising because GPCRs can be coupled to several G-proteins, and activate different second messengers and downstream signaling pathways. In the case of ALX, the variety of peptide agonists identified, in addition to $LXA_4$, makes the analysis of signaling more complex. The emerging scenario is that ALX is a peculiar receptor capable of switching responses from anti- to proinflammatory in relation to the activating agent. Thus, $LXA_4$, ATL, and some peptides trigger potent anti-inflammatory signaling and bioactions, whereas a number of endogenous or disease-associated peptides stimulate proinflammatory events. The question is: Which event predominates and when? One important consideration is that $LXA_4$ and ATL are the most potent ALX agonists known. They are active in the nanomolar range, as opposed to the majority of peptides,

**TABLE 3**
**Characteristics of ALX Agonists**

| Ligand | ALX | |
|---|---|---|
| | $EC_{50}$ | $K_d$ |
| $LXA_4$ and ATL | | 1.7 n*M* |
| Bacterial-derived peptides | | |
| fMLF | | >1 µ*M* |
| Hp2-20 | 300 n*M* | |
| Nonformylated peptides | | |
| Annexin 1 | | >900 n*M* |
| D2D3 uPAR fragment | 0.1 n*M* | 83 n*M* |
| SAA | | 45 n*M* |
| $A\beta_{42}$ | 1.5 µ*M* | |
| $PrP_{106-126}$ | 10 µ*M* | |
| HN | 3.5 µ*M* | |
| MHC binding peptide | | 1 n*M* |
| LL-37 | 5 µ*M* | |
| CKβ8-1 | 1 µ*M* | |
| F2L | >1 µ*M* | |
| Temporin A | ~2 µ*M* | |
| HIV-envelope peptides | | |
| T20 | | |
| T21 | 500 n*M* | |
| N36 | 10 µ*M* | |
| F peptide | 10 µ*M* | |
| V3 peptide | 1.5 µ*M* | |
| Synthetic peptides | | |
| WKYMVm | 75 p*M* | |
| MKK-1 | 2 n*M* | |
| WRW | 1–10 µ*M* | |

which activate ALX at micromolar concentrations. Moreover, transgenic mice overexpressing ALX in myeloid cells are more resistant to zymosan-induced peritonitis and produce less inflammatory exudates[67]. Thus, it is likely that, *in vivo*, the anti-inflammatory properties of ALX may predominate, at least during physiological host-responses. This does not exclude that in pathological conditions, the accumulation of disease-related peptides, i.e., SAA, A$\beta_{42}$, gp120, may overt $LXA_4$ biosynthesis and trigger proinflammatory ALX signaling. The Janus-like aspect of ALX can be synthesized by the observation that in the brain, ALX can be activated by A$\beta_{42}$[87], which damages neuronal cells and is related to AD, but also by HN, which protects neuronal cells from damage by A$\beta_{42}$[76]. Disease may occur when equilibrium is broken.

## Intracellular Calcium

Increases in $[Ca^{2+}]_i$ are induced by $LXA_4$ and ATL, as well as by peptide agonists, both in myeloid cells and in ALX transfected cells (Fig. 5). In monocytes, $LXA_4$-induced $[Ca^{2+}]_i$ increase derives from both

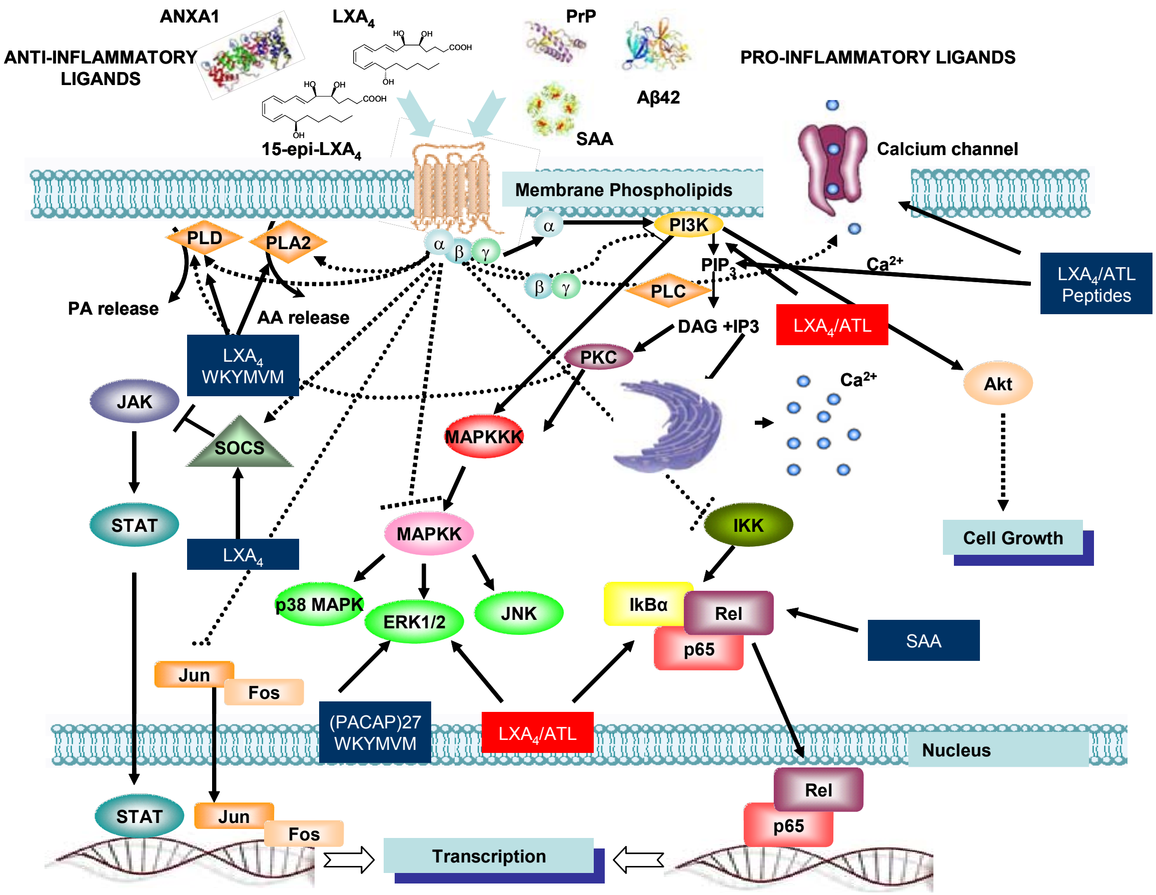

**FIGURE 5**. Intracellular signaling of ALX. Positive agonists are in the blue boxes, inhibitors are in the red boxes (see text for details).

intracellular mobilization and external influx[28], suggesting that ALX is coupled with membrane $Ca^{2+}$ channels and with $IP_3$ generation. Indeed, accumulation of $IP_3$ in $LXA_4$-stimulated COS-1 cells cotransfected with mouse ALX and G$\alpha$16 has been documented[66]. $LXA_4$ also induced $[Ca^{2+}]_i$ increase in ALX-expressing human respiratory epithelial cells[100]. $[Ca^{2+}]_i$ transients were suppressed by thapsigargin, but were left unchanged by EGTA, indicating that release from thapsigargin-sensitive intracellular stores is mainly involved. In these cells, $LXA_4$ induced sustained $Cl^-$ secretion.

### AA Release

$LXA_4$ stimulates release of AA in PMN, differentiated HL-60 cells, and also in ALX-transfected CHO cells[53,55]. In ALX-transfected cells, AA release was maximal after three to five exposures to $10^{-9}$ *M* $LXA_4$. AA release in PMNs was also induced by the peptide WKYMVM[101] (Fig. 5).

### Phospholipase D

In PMN and retinoic acid–differentiated HL-60 cells, $LXA_4$ activates phospholipase D (PLD) in a biphasic mode, with a first peak of activity at $10^{-9}$ *M* and a second peak at $10^{-7}$[53]. Interestingly, the first peak of activity was inhibited by PTX, whereas the second was blocked by staurosporine, indicating that in addition to G-proteins, protein kinase C (PKC) may be involved in ALX signaling. A PKC-dependent activation of PLD was observed in PMN stimulated with the ALX peptide agonist WKYMVM[101] (Fig. 5).

### Polyisoprenyl Phosphate

In PMNs, $LXA_4$ and ATL reversed the decrease in presqualene diphosphate levels evoked by $LTB_4$. Thus, they promoted the presqualene diphosphate anti-inflammatory pathway, documented by inhibition of superoxide anion generation and PLD activity[102].

### Kinases

The ERK pathway can be either activated or inhibited following engagement of ALX agonists. ERK phosphorylation was induced in PMNs by the neuropeptide pituitary adenylate cyclase-activating polypeptide (PACAP)27, in conjunction with CD11b up-regulation and chemotactic migration[103]. WKYMVM also induced ERK phosphorylation in PMNs[101]. This was associated with c-Jun $NH_2$-terminal kinase phosphorylation and $O_2^-$ generation. On the contrary, nanomolar concentrations of ALTa inhibited the anti-CD3 antibody-mediated activation of ERK and secretion of TNF-α in human T lymphocytes[31]. In human lung fibroblasts, $LXA_4$ down-regulated ERK as well as PI3K/Akt activation by connective tissue growth factor, resulting in reduced proliferation[104] (Fig. 5). The involvement of ALX was confirmed with overexpression experiments. Thus, proinflammatory ALX agonists may turn ERK on, whereas anti-inflammatory ligands, such as ATLa, turn it off. This clearly illustrates the bimodal function of ALX.

Protein kinase A (PKA) activation occurs during $LXA_4$-induced phagocytosis of apoptotic neutrophils by monocyte-derived macrophages. This key mechanism of inflammation resolution is associated with rearrangement of F-actin filaments, also involving the small GTPases RhoA and Rac[105]. Along these lines, ATLa inhibited VEGF-induced SAPK/p38 activation and focal adhesion kinases (FAK) clustering in endothelial cells, blocking cell growth, adhesion, and migration[106]. Thus, ALX controls proangiogenic responses that may be relevant in a variety of pathological conditions, including neoplasia, diabetic retinopathy, myocardial infarction, and atherosclerosis.

### NF-κB

NF-κB is a key regulator of the inflammatory response. A number of reports have documented NF-κB modulation by ALX agonists. Again, it is striking to denote NF-κB activation by proinflammatory ALX agonists and inhibition by anti-inflammatory agonists, confirming the dual immunoregulatory potential of this receptor. In human leukocytes, $LXA_4$ and ATL markedly inhibited NF-κB and activator protein-1 (AP-1) induced by LPS, resulting in reduced IL-8 mRNA expression and protein secretion[22]. Likewise, in a model of inflamed gastrointestinal epithelium, microarray gene expression profile analysis revealed that ALX activation by $LXA_4$ attenuated NF-κB–induced expression of several proinflammatory genes in response to *Salmonella typhimurium*[47]. A direct evidence of the inhibitory action of $LXA_4$-activated ALX on NF-κB activity was obtained using HeLa cells transfected with an ALX cDNA[47]. Along these lines, using CHO cells stably transfected to express human ALX together with the promoter of human IL-8, Sodin-Semrl et al. demonstrated that the NF-κB pathway is the main mechanism involved in $LXA_4$-elicited down-regulation of this cytokine[107]. Consistently, using embryonic HEK293 cells expressing both the recombinant human ALX and the NF-κB responsive element (NRE)-luciferase vector, Devchand et al. assessed $LXA_4$-mediated inhibition of TNFα-induced NF-κB activation[67]. On the other side, SAA activated NF-κB in human PMNs, inducing IL-8 secretion. This effect was better observed in HeLa cells overexpressing ALX[86] (Fig. 5).

### SOCS-2

$LXA_4$ up-regulates SOCS-2 expression, *in vivo* and *in vitro*[33] (Fig. 5). This observation highlights the immunoregulatory properties of $LXA_4$ in host defense. In fact, SOCS-2–deficient mice have an exuberant immune response, with an uncontrolled production of cytokines and leukocyte infiltration, a greater mortality, and impaired bacteria clearance[33].

## ADDITIONAL $LXA_4$ RECOGNITION SITES

The possibility that $LXA_4$ may interact with multiple intracellular sites was postulated in initial binding studies with radiolabeled $LXA_4$. Specific binding was in fact localized in PMN granule- and nuclear-enriched fractions[52]. It has been reported that $LXA_4$ binds to the transcription factor termed Ah receptor (AhR) in Hepa-1 cells[108]. The specific binding is associated with transition of AhR into an active dioxin response element (DRE)-binding state and concentration-dependent increment in mRNA of *CYP1A1*, which is a recognized molecular target of AhR[108]. Interestingly, AhR is required for $LXA_4$-induced up-regulation of SOCS-2 and inhibition of IL-12 release in mouse splenic dentritic cells[33].

On the other hand, $LXA_4$ interaction with CysLT receptors has been clearly documented. Early studies showed $LXA_4$ competition for radiolabeled $LTD_4$ in mesangial and endothelial cells[109] as well as displacement of radiolabeled $LXA_4$ by molar excess of $LTD_4$ or of a CysLT antagonist[53]. These results were confirmed by more recent investigation showing that ATLa and $LTD_4$ competed with equal affinity ($IC_{50}$ = ~0.7 nmol/l) for $[^3H]$-$LTD_4$ binding to recombinant endothelial-type $CysLT_1$ receptor expressed in COS-7 cells[54]. Consistently, ATLa and $LTD_4$ competed for $[^3H]$-ATLa binding ($IC_{50}$ = ~0.1 and 0.9 nmol/l, respectively). Displacement of radiolabel was not observed with 6*S*-$LXA_4$, indicating that the *rectus* chirality at carbon 6, shared by $LTD_4$ and ATLa, is crucial for $CysLT_1$ receptor recognition. Thus, competition at the receptor level represents the main mechanism of $LXA_4$ counter-regulatory actions on $LTD_4$-induced inflammatory events, such as vascular leakage. Therefore, $LXA_4$ may be also regarded as a $CysLT_1$ antagonist. $LXA_4$ interactions with CysLT receptors may be, however, more complex. In mesangial cells, $LXA_4$ counteracts $LTD_4$-induced proliferation and activates ERK and p38[110]. However, activation of ERK was insensitive to PTX, whereas activation of p38 was blocked by PTX and by the $LTD_4$ receptor antagonist SKF 104353. Together, these findings suggest that additional $LXA_4$ recognition sites may exist.

$LXB_4$ shares a number of anti-inflammatory bioactions with $LXA_4$. However, it does not compete for $^3H$-$LXA_4$ binding, both with PMNs or ALX-transfected cells. This may be an indication that $LXB_4$ recognizes receptor(s) other than ALX, yet to be discovered.

## CONCLUSIVE REMARKS

LXs are emerging as key endogenous stop signals of inflammation. The identification of the $LXA_4$ receptor, ALX, has represented a significant advance for the understanding of the molecular mechanisms involved in LX anti-inflammatory actions. The great interest in ALX is justified by the fact that ALX appears to regulate host-response and resolution. A number of anti-inflammatory mediators, endogenously generated and pharmacologically induced (aspirin/ATL, corticosteroids/annexin1), appear to signal through this receptor. ALX, however, can also convey proinflammatory signals, *in vitro*, when activated by disease-related peptides (neurodegeneration, HIV infection). Animal studies and analysis of ligand affinity indicate that under physiological circumstances, the anti-inflammatory properties of ALX are predominant. Less is known on ALX in human disease. Are altered expression and/or function of this receptor associated with the development of inflammatory disorders? Given the documented possibility to design ALX agonists with selective anti-inflammatory activity, this receptor may represent a molecular target to be exploited for innovative pharmacological approaches to inflammation-based disorders.
